# Supplementary material for: About the Transient Effects of Synthetic Amorphous Silica: An In Vitro Study on Macrophages
Source: Int J Mol Sci. 2022 Dec 22;24(1):220. doi: 10.3390/ijms24010220 (PMC9820141; doi:10.3390/ijms24010220)
Supplement: Supplementary file 1 [file ijms-24-00220-s001.zip › Control experiments V2.pdf]

Control experiment 1: viability measurements

Material and methods

J774 cells were seeded in 12 well plates at 500,000 cells/ml, left for 24 hours and then treated with silica at the designated concentrations for 24 hours. The cells were resuspended in the medium, 2/3 of the suspension was submitted to a viability test by the propidium iodide method described in the article, while 1/3 was submitted to a manual viability test by eosin Y exclusion (1 mg/ml final concentration) and numeration in a Malassez counting cell. The experiment was carried out in quadruplicate.

Results (expressed as mean±standard deviation of viable cells)

Condition	Viability by propidium iodide	Viability by eosin
CTL	94+4±0.5	97.2±1.1
SC-20µg/ml	84.8±2.0	85.7±4.7
MC-20µg/ml	67.4±5.9	68.6±0.7
SPr-100µg/ml	72.1±0.9	69.5±5.9
MPr-100µg/ml	72±2.6	75±1.5
SPy-35µg/ml	65.4±2.8	74.5±2.8

Conclusions

Except for the condition with pyrolytic silica, the two completely independent viability assays gave similar results, indicating no gross artifact arising in the propidium iodide method

Control experiment 2: fluorophore leakage from latex beads used for the phagocytosis experiments (long exposure)

Material and methods

J774 cells were seeded in 12 well plates at 500,000 cells/ml and left to adhere for 24 hours. The cells were then exposed to FITC-latex beads (Sigma L4655) at a concentration of 20μg/ml for 3 hours to ensure complete phagocytosis of the beads. The culture medium was removed, the cell layer rinsed twice with PBS, and then lysed in 400μl of 10mM Hepes pH 7.5. The lysate was recovered, and its fluorescence measured on a DeNovix QFX fluorimeter (excitation 470nm, emission 514 nm). The lysate was then recovered and centrifuged at 15,000g for 30 minutes to pellet the beads. The supernatant was recovered and its fluorescence measured again.

Results

Fluorescence (arbitrary units) mean±standard deviation	Buffer	Unexposed cells	Beads-exposed cells
Before centrifugation	112	219±3	16167±1502
After centrifugation		195±4	243±12

Conclusions

By substracting the values obtained from unexposed cells from the values obtained from exposed cells, it is possible to calculate the total fluorescence due to the beads and the one due to leakage (i.e. the one

remaining after centrifugation). It appears that the fluorophore leakage represents only 0.3% of the total fluorescence after an 18 hours exposure.

Control experiment 3: fluorophore leakage from latex beads used for the phagocytosis experiments (short exposure)

Material and methods

J774 cells were seeded in 12 well plates at 500,000 cells/ml and left to adhere for 24 hours. The cells were then exposed to FITC-latex beads (Sigma L4655) at a concentration of 20μg/ml for 3 hours, to mimic a phagocytosis experiment. The culture medium was removed, the cell layer rinsed twice with PBS, and then lysed in 400μl of 10mM Hepes pH 7.5. The lysate was recovered, and its fluorescence measured on a DeNovix QFX fluorimeter (excitation 470nm, emission 514 nm). The lysate was then recovered and centrifuged at 15,000g for 30 minutes to pellet the beads. The supernatant was recovered and its fluorescence measured again.

Results

Fluorescence (arbitrary units) mean±standard deviation	Buffer	Unexposed cells	Beads-exposed cells
Before centrifugation	107	163±5	23302±1549
After centrifugation	107	136±2	567±102

Conclusions

By substracting the values obtained from unexposed cells from the values obtained from exposed cells, it is possible to calculate the total fluorescence due to the beads and the one due to leakage (i.e. the one remaining after centrifugation). It appears that the fluorophore leakage represents only 0.2% of the total fluorescence after a 3 hours exposure.

Control experiment 4: interference of silica with cytokine dosage

Complete culture medium was supplemented with 100μg/ml of each amorphous silica and incubated for 24 hours in a cell culture incubator. A known concentration of cytokines (165 pg/ml final concentration of Interleukin-6 and 189 pg/ml of TNF-alpha) was then added and the medium returned to the cell incubator for 6 hours. The medium was then centrifuged (200g, 5 minutes, the supernatant collected and frozen at -20°C. The cytokine dosage was performed as described in the article, on independent triplicate points. The following values (expressed as mean±standard deviation) were obtained.

Interleukin-6	Measured value	Ratio over control value
Control (medium +cytokine)	174.4±12.9	1
SC	130.6±17	0.75
MC	143.9±26.8	0.82
SPr	153.7±2.5	0.88
MPr	166.6±15.1	0.96
SPy	160.1±32.2	0.92

TNF-alpha	Measured value	Ratio over control value
Control (medium +cytokine)	191.4±14.7	1
SC	169±35.3	0.88
MC	188.6±51.8	0.98
SPr	188.2±6.7	0.98
MPr	170.8±13.3	0.89
SPy	215.8±48.6	1.13

Conclusions

The interference caused by the presence of silica in the cytokine dosage was in the ±10-25% range, which indicates at worst a minor interference in the cytokine dosage.
