# Supplementary material for: About the Transient Effects of Synthetic Amorphous Silica: An In Vitro Study on Macrophages
Source: Int J Mol Sci. 2022 Dec 22;24(1):220. doi: 10.3390/ijms24010220 (PMC9820141; doi:10.3390/ijms24010220)
Supplement: Supplementary file 1 [file ijms-24-00220-s001.zip › SupTable S1.pdf]

Supplementary Table S1

Results of the ANOVA+Tukey’s pairwise test
p value of the ANOVA: 0.00182

Tukey’s Q values are reported below the diagonal, and p(same) values are reported above the diagonal

	SC	MC	SPr	MPr	SPy
SC		0.769	0.124	0.542	0.030
MC	1.651		0.021	0.993	0.005
SPr	3.816	5.467		0.011	0.886
MPr	2.230	0.578	6.046		0.003
SPy	5.107	6.758	1.291	7.336	
